# A multi-nozzle nebuliser does not improve tissue drug delivery during PIPAC

**DOI:** 10.1007/s00464-024-11172-4

**Published:** 2024-08-19

**Authors:** Yaroslaw Sautkin, Juergen Weinreich, Marc André Reymond

**Affiliations:** 1 National Center for Pleura and Peritoneum, University Hospital Tübingen, Hoppe-Seyler-Straße 3, 72076 Tübingen, Germany; 2 Department of General, Visceral and Transplant Surgery, University Hospital Tübingen, Hoppe-Seyler-Straße 3, 72076 Tübingen, Germany

**Keywords:** Pressurised intraperitoneal aerosol chemotherapy—PIPAC, Peritoneum, Peritoneal metastasis, Intraperitoneal drug delivery, Nebuliser

## Abstract

**Background:**

Multi-nozzle nebulisers for pressurised intraperitoneal aerosol chemotherapy (PIPAC) are implemented in clinical practice to improve the homogeneity of tissue drug delivery. Nonetheless, the advantages of such devices over one-nozzle nebulisers have not been demonstrated thus far. In this study, we compared the performance of multi- and one-nozzle nebulisers by conducting physical and ex vivo pharmacological experiments.

**Methods:**

The one-nozzle nebuliser Capnopen® and the multi-nozzle nebuliser were the subjects of this study. In physical experiments, the aerosol droplet size was measured by laser diffraction spectroscopy. Spatial spray patterns were depicted on blotting paper. Pharmacological experiments were performed on the enhanced inverted bovine urinary bladder model, demonstrating real-time tissue drug delivery, aerosol sedimentation and homogeneity of doxorubicin and cisplatin tissue distribution.

**Results:**

The multi-nozzle nebuliser had a sixfold greater aerosolisation flow and a threefold greater angle of aerosolisation than Capnopen®. The aerosol particle size and distribution range were higher than that of Capnopen®. Spray patterns on blotting paper were more extensive with the multi-nozzle nebuliser. Real-time tissue drug delivery with the multi-nozzle nebuliser was over 100 ml within 1 min, and the aerosol sedimentation was 48.9% ± 21.2%, which was not significantly different from that of Capnopen®. The doxorubicin and cisplatin tissue concentrations were greater with Capnopen®. Although there was no significant difference in the homogeneity of doxorubicin distribution between the two devices, the homogeneity of cisplatin distribution was significantly higher with Capnopen®.

**Conclusion:**

The multi-nozzle PIPAC nebuliser did not fulfil expectations. Even though the surface spray patterns were broader with the multi-nozzle nebuliser, the tissue drug homogeneity and concentration were greater with Capnopen®.

**Graphical Abstract:**

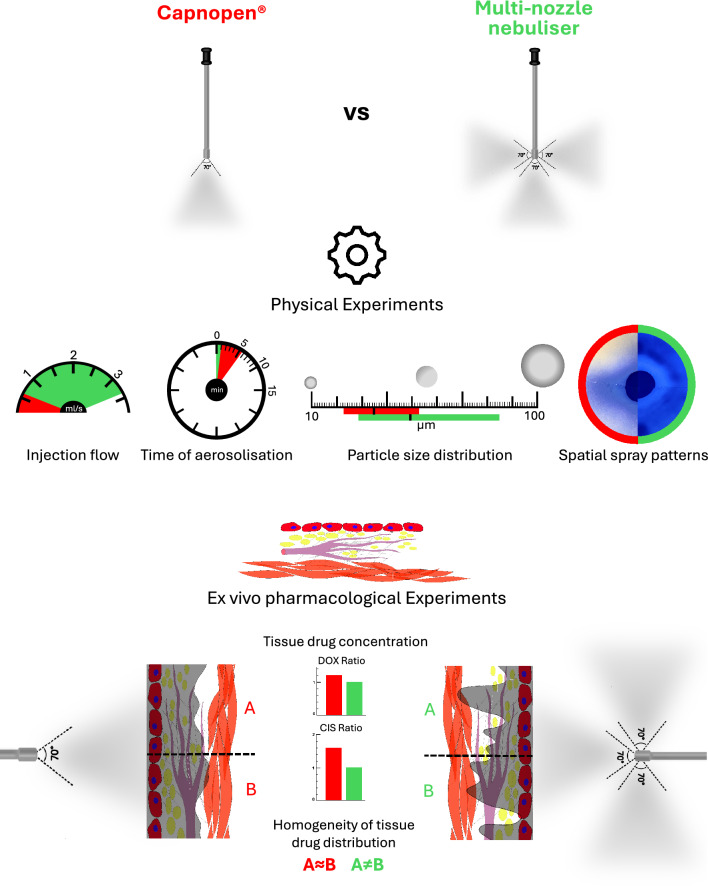

Homogeneous tumour exposure to anticancer substances at a cytotoxic concentration determines an individual’s response to therapy. In the treatment of peritoneal surface malignancies, hydrophilic drugs are mainly applied and characterised by a low tissue penetration depth and inhomogeneous distribution [1]. For instance, the depth of doxorubicin penetration after intraperitoneal liquid chemotherapy is 4–6 cell layers [2], and that of cisplatin is 35–50 µm after HIPEC [3].

Pressurised intraabdominal aerosol chemotherapy (PIPAC) is a minimally invasive, high-volume drug delivery system offering higher tissue drug concentration [4], deeper drug penetration [5] and more homogeneous tissue drug distribution than conventional lavage [6, 7] or intravenous chemotherapy [8]. Furthermore, the tissue drug delivery with PIPAC exceeds 100 ml within 6 min [9].

Despite the advantages of PIPAC, the tissue drug distribution remains heterogeneous. As the homogeneity of drug distribution during PIPAC mainly depends on the nebuliser, there have been numerous attempts to improve the technology. For instance, PIPAC combined with electrostatic precipitation (ePIPAC) considerably increased the homogeneity of drug delivery with a depth of tissue drug penetration greater than 5000 µm [9]. Another attempt to increase surface drug exposure was to add a conical rotation moment to a nebuliser, which led to better tissue drug distribution [10]. Alternatively, a higher angle of aerosolisation and broader tissue drug exposure were reached by multi-nozzle nebulisers, e.g. the multi-nozzle nebuliser (Capnopharm GmbH, Tübingen, Germany) or Quattrojet® (REGER Medizintechnik GmbH, Villingendorf, Germany). However, there are no proper preclinical data showing the advantages of multi-nozzle devices in drug delivery compared to a standard one-nozzle nebuliser, e.g. Capnopen®.

This study provides preclinical data on drug delivery by comparing the multi-nozzle nebuliser with the one-nozzle nebuliser Capnopen® (Fig. 1).

**Fig. 1:**
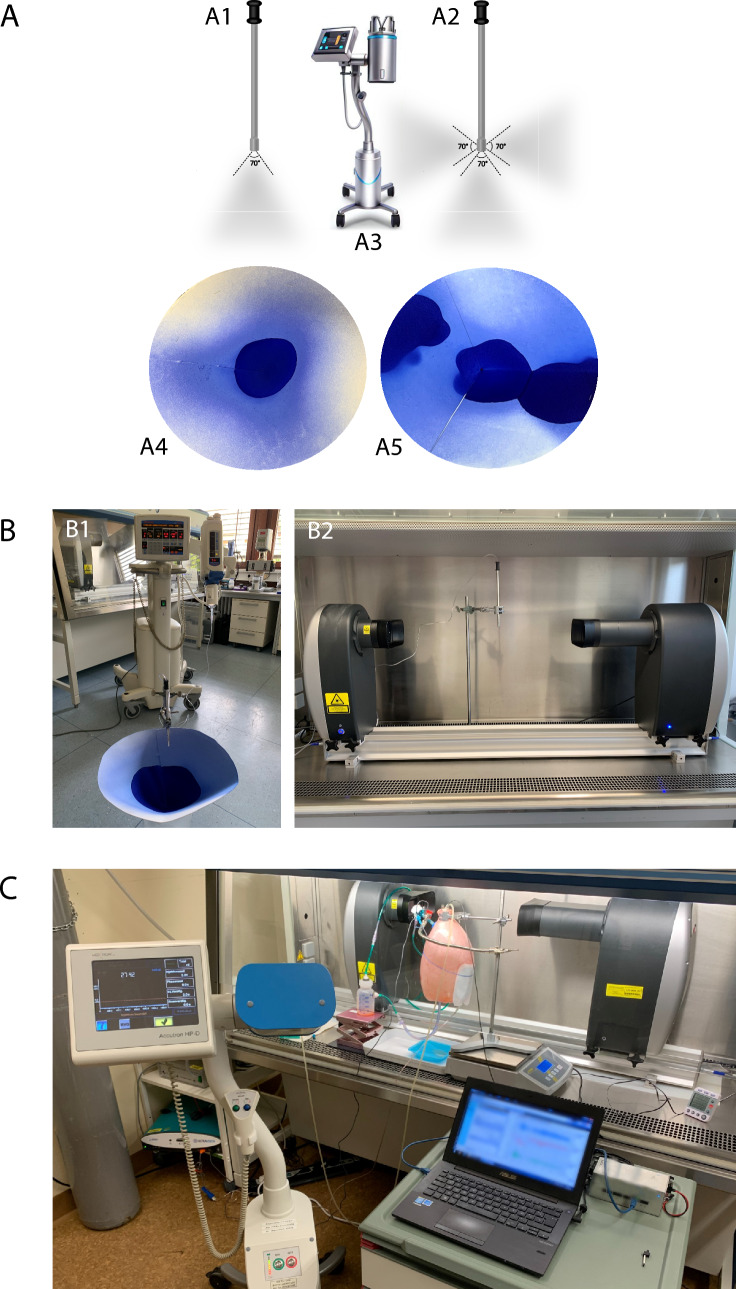
Materials and methods. **A**: PIPAC technological components. A1—Standard one-nozzle nebuliser Capnopen®, A2—Multi-nozzle nebuliser, A3—Angio-injector, A4—Aerosolisation patterns with Capnopen® and A5—multi-nozzle nebulisers on a volumetric blotting paper model. **B**: Setup of the physical experiments. B1—Volumetric blotting paper model for evaluating the surface spray patterns, B2—Granulometry of the aerosol particles by laser diffraction spectroscopy [9]. **C**: Setup of the ex vivo enhanced inverted bovine urinary bladder model (eIBUB) [13]

## Methods

### Study design

**Fig. 2:**
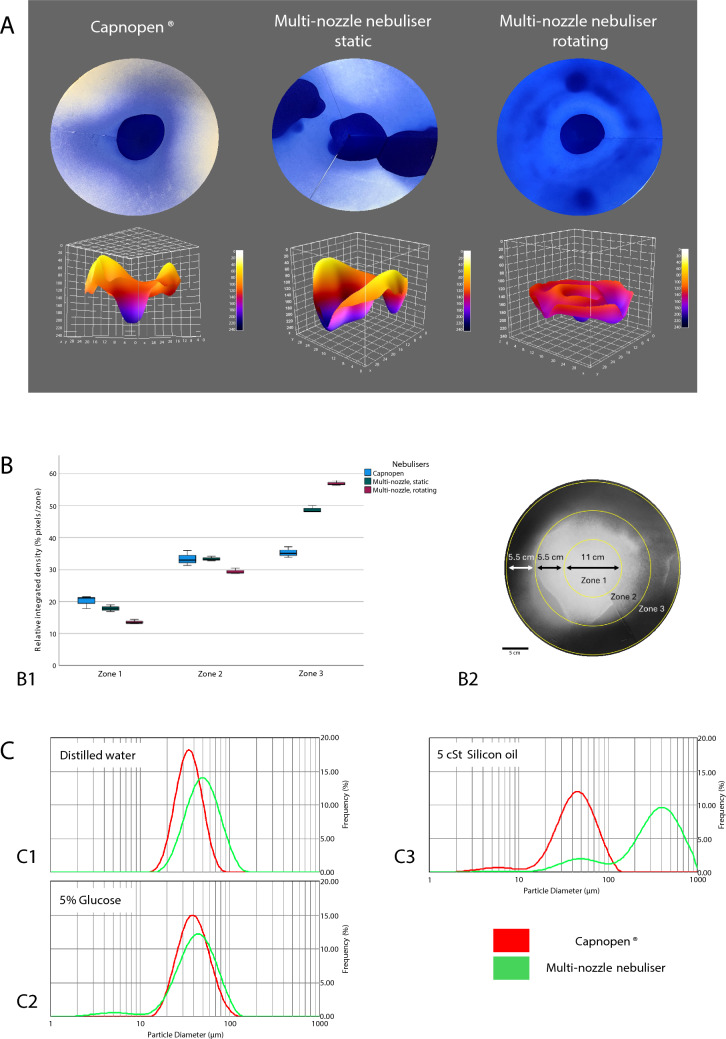
Results of the physical experiments. **A**: 3D evaluation of surface ink cover after aerosolisation with the one-nozzle nebuliser Capnopen® vs the static and rotating multi-nozzle nebuliser on a volumetric blotting paper model. The surface ink cover was more extensive with the multi-nozzle nebuliser and further enhanced by rotation. **B**: Distribution of blue ink among three concentric zones on the volumetric blotting paper model after aerosolisation with the Capnopen® and multi-nozzle nebulisers. B1—Number of stained pixels per zone, B2—Zone topography of the blotting paper model. The difference between zones 2 and 3 was less pronounced with the Capnopen® nebuliser. The surface ink cover in zone 3 was greater with the multi-nozzle nebuliser. **C**: Aerosol particle size distribution with the Capnopen® nebuliser (red line) and the multi-nozzle nebuliser (green line). C1—Distilled water, C2–5% glucose, C3–5 cSt silicon oil. The aerosol particle size and distribution range were lower with the Capnopen® nebuliser than with the multi-nozzle nebuliser

**Fig. 3:**
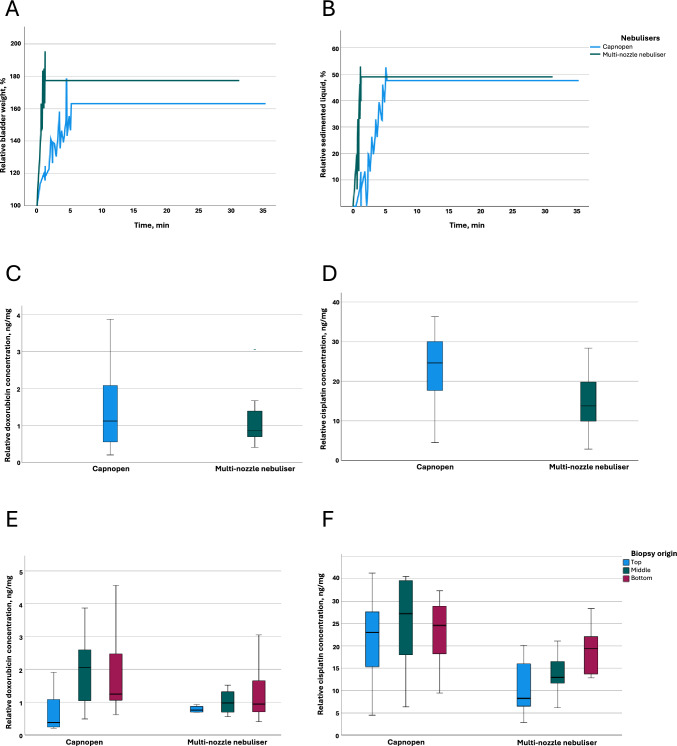
Results of the ex vivo pharmacological studies with enhanced inverted bovine urinary bladder model (eIBUB). **A**: The relative eIBUB weight during drug aerosolisation and at steady state. The duration of aerosolisation with the one-nozzle nebuliser Capnopen® was 6 min, and that with the multi-nozzle nebuliser was 1 min. The weight gain of the target tissue was greater than 160% in both groups (one-way ANOVA, *p* > 0.05). **B**: Percentage of liquid sedimentation after aerosolisation with Capnopen® and the multi-nozzle nebuliser. The median sedimented liquid volume was less than 50% in both groups (one-way ANOVA, *p* > 0.05). Aerosol sedimentation occurred mainly during aerosolisation. **C**: The total doxorubicin tissue concentration after PIPAC with Capnopen® and the multi-nozzle nebuliser (one-way ANOVA, *p* > 0.05). **D**: The total cisplatin tissue concentration after PIPAC with Capnopen® and the multi-nozzle nebuliser (one-way ANOVA, *p* < 0.001). **E**: The doxorubicin tissue concentrations in the top, middle and bottom eIBUB tissue biopsies after PIPAC with Capnopen® and the multi-nozzle nebuliser (one-way ANOVA, *p* > 0.05). **F**: The cisplatin tissue concentrations in the top, middle and bottom eIBUB tissue biopsies after PIPAC with Capnopen® and the multi-nozzle nebuliser (one-way ANOVA, *p* < 0.05 between two groups in the top and middle tissue biopsies)

The performance of the multi-nozzle nebuliser was compared with that of the current clinical reference one-nozzle Capnopen® via physical (Fig. 2) and ex vivo pharmacological experiments (Fig. 3). In physical experiments, the granulometry of aerosol particles and spatial spray patterns were analysed. Ex vivo, tissue drug delivery and homogeneity of drug distribution were evaluated. We assumed that a greater number of nozzles might increase the angle of aerosolisation and, thus, the tissue drug delivery and the homogeneity of tissue drug distribution.

### Ethical and regulatory approvals

According to German law and European regulations, no ethical approval was required for the physical or ex vivo experiments [11, 12]. No animals were sacrificed for scientific purposes.

### Occupational health and safety

The experiments were performed in the laboratory with continuous room airflow on a cytostatic-approved safety bench wearing protective clothes. Air and surface contamination with cytotoxic drugs was regularly investigated. The laboratory personnel were trained for manipulating cytotoxic drugs and were certified for “Good Laboratory Practice” (GLP) and “Good Scientific Practice” (GSP).

### Drugs

Doxorubicin (Cell Pharm® GmbH, Bad Vilbel, Germany) and cisplatin (Teva® GmbH, Ulm, Germany) are the most often applied combinations of cytostatics during PIPAC. A total of 2.7 mg of doxorubicin was dissolved in 50 ml of 0.9% NaCl, and 13.5 mg of cisplatin was dissolved in 150 ml of 0.9% NaCl. Both drugs were aerosolised at room temperature.

### Nebulisers

Capnopen® (Capnopharm GmbH, Tuebingen, Germany) is an MDR class IIb certified device for aerosolising chemotherapies, nanoparticles, DNA, RNA, viruses and immunotherapy. A multi-nozzle nebuliser (Capnopharm GmbH, Tuebingen, Germany) is a prototype containing three spray nozzles that are technically similar to those in Capnopen®. Both nebulisers have an operation pressure of 11–20 bars and can be used with any angio-injector approved for PIPAC. The Capnopen® nozzle is located along the nebuliser shaft with an angle of aerosolisation of 70°, whereas the multi-nozzle nebuliser possesses three nozzles, two at the side of the nebuliser shaft and one along the nebuliser axe. The angle of aerosolisation for each of the three nozzles is 70°. The angle between the side nozzles is 180°, and the angle between the side and axial nozzles is 90° (Fig. 1A1, A2).

Both nebulisers were used in this study and operated with an Accutron HP-D angio-injector (Medtron AG, Saarbruecken, Germany) under a recommended pressure of 11–20 bars utilising a high-pressure line. Multiple manual axial rotations of 360° were added to the multi-nozzle nebuliser to extend the surface exposure.

### PIPAC

The performance of both nebulisers was evaluated via physical and ex vivo experiments during a standard PIPAC procedure comprising 6 min of aerosolisation and a 30 min steady-state phase. In the physical experiments, the granulometry of the aerosol particles and spatial aerosolisation patterns were analysed (Fig. 1B1, B2), and in the ex vivo experiments, real-time tissue drug delivery and the homogeneity of tissue drug distribution were demonstrated with enhanced inverted bovine urinary bladder model (eIBUB) (Fig. 1C).

### Physical experiments

#### Granulometry of the aerosol particles

In our experience, the size of aerosol particles modifies the tissue drug distribution. The smaller and more homogeneous the particles are, the greater the distribution homogeneity in the target tissue is. The aerosol particle size distribution (MAD) was measured by laser absorption spectroscopy with Spraytec™ (Malvern Panalytical GmbH, Kassel, Germany), while aerosolising 50 ml of distilled water, 5% glucose and 5 cSt silicon oil at room temperature, three measurements were taken with each solution and nozzle. Both nebulisers were located 10 cm above the laser beam (Fig. 1B2).

#### Aerosolisation patterns on blotting paper

The homogeneous exposure of the target tissue to antitumour drugs determines an individual’s therapeutic response. The spray distribution patterns of the multi-nozzle and Capnopen® nebulisers were evaluated on conic folded blotting paper (Fig. 1A4, A5, B1). Each nebuliser was located at the level of the cone base, and 50 ml of blue ink (Pelikan Ink 4001, Royal Blue, Pelikan Group GmbH, Hannover, Germany) was aerosolised, followed by photo documentation. The relative integrated density of the staining was calculated with ImageJ™ (National Institutes of Health, Bethesda, Maryland, USA) among three circle zones (Fig. 2B2). The performance of the multi-nozzle nebuliser was demonstrated with and without axial rotations and compared with that of Capnopen®.

### Ex vivo experiments on eIBUB model

The ex vivo eIBUB model was proposed in 2019 by Sautkin et al. for pharmacological distribution studies on the peritoneum treated with PIPAC or ePIPAC [13]. The bovine urinary bladder is entirely lined with mesothelial cells and has a volume of 2–5 L, similar to that of the human abdominal cavity. By inverting the organ inside out, with the serosa on the inside and the mucosa on the outside, drugs can be applied intraluminally to investigate the peritoneal drug concentration and depth of drug penetration. Based on the eIBUB design, the target tissue is only exposed to aerosols and not to sedimented liquid, and any tissue weight gain can be measured in real time, indicating tissue drug delivery. Here, we conducted PIPAC with the multi-nozzle and Capnopen® nebulisers on the eIBUB model (Fig. 1C). The multi-nozzle nebuliser was manually rotated during aerosolisation to increase surface drug exposure.

#### Model setup

The eIBUB model utilises the principle of communicating vessels with an inverted bovine urinary bladder connected to an airtight plastic cup where a sedimented liquid collects. The spray nozzle is introduced intraluminally through a trocar at the bladder neck and connected to a high-pressure angio-injector through a line. The bladder and plastic cup are supplied with CO_2_ at 15 mmHg and positioned on a scale to observe the weight changes (Fig. 1A3, C). Two groups of three bladders were assigned for PIPAC with the multi-nozzle nebuliser and Capnopen® nebuliser. At the end of each experiment, three tissue biopsies were taken from the top, middle and bottom of each bladder to measure the tissue drug concentration, defining the homogeneity of drug distribution.

#### Real-time measurements of tissue drug delivery and aerosol sedimentation

Drug delivery into the target tissue determines the efficacy of treatment. Tissue drug delivery during the aerosolisation and steady-state phases of PIPAC was measured with the eIBUB model. The increase in bladder weight demonstrated drug delivery into the tissue and was measured with a scale (FCB12K1, Kern GmbH, Balingen, Germany) and recorded automatically in Microsoft Excel (Microsoft Office, Microsoft Corp., Washington, USA) by balance connection software (Kern SCD-4.0, connection via RS-232, Kern & Sohn GmbH, Balingen, Germany).

Aerosol sedimentation also characterises the efficacy of drug delivery. The higher the volume of sedimented aerosol, the lower the tissue drug delivery is. The volume of liquid collected in an airtight plastic cup represents aerosol sedimentation and was weighed with a scale (PCB 600-2, Kern GmbH, Balingen, Germany).

#### Preanalytical sample preparation

At the end of each experiment, three tissue biopsies were taken from the top, middle and bottom of each bladder to measure the doxorubicin and cisplatin tissue concentrations, and the samples were preserved at − 80 °C. Sample preparation began with probe lyophilisation for 24 h (Speedevac KF-2-110; H. Saur Laborbedarf, Reutlingen, Germany), followed by weighing. Afterwards, the probes were placed in 2 ml PowerBead™ tubes (QIAGEN GmbH, Hilden, Germany) filled with 1.5 ml of distilled water and homogenised for 2 h with TissueLyser LT™ (QIAGEN GmbH, Hilden, Germany) at 50 Hz. Finally, the samples were centrifuged at 11,000 rpm and frozen at − 80 °C until analysis.

#### Measurements of tissue drug homogeneity

The homogeneity of tissue drug distribution was analysed based on the doxorubicin and cisplatin tissue concentrations in the top, middle and bottom biopsies of each bladder and among the bladders. The tissue drug concentration was measured in an external GLP-certified laboratory (Medizinisches Versorgungszentrum Dr. Eberhard & Partner, Dortmund, Germany). Doxorubicin was analysed by HPLC (Waters Fluorescence Detector 2475, Waters Inc., Milford, MA, USA). The lower limit of quantification (LLoQ) was 5 ng/ml, and preanalytical validation showed a linear range of measurements in a 5% glucose matrix between 0.1 and 10,000 µg/ml doxorubicin with no influence of organic matrices. The cisplatin concentration was analysed by atomic absorption spectroscopy (AAS; ZEEnit P 650, Analytic Jena AG, Jena, Germany). The LLoQ for cisplatin was 80 ng/ml. Preanalytical validation confirmed a linear range of measurements in a 5% glucose matrix between 0.1 and 100 µg/ml platinum and established no influence of organic matrices.

### Data and statistical analysis

The data were collected and processed according to the internal SOP guided by the GSP. The experimental data were stored on the clinic server. The statistical data were processed with SPSS™ (IBM, NY, USA).

- Descriptive statistics. The central tendency is shown by the median and mean, and variability is demonstrated by the standard deviation and 95% confidence interval.
- Comparative statistics. The obtained data were not normally distributed. Comparisons among groups were performed using nonparametric tests, such as post hoc tests and one-way ANOVAs, and ≤ 0.05 indicates statistical significance.

## Results

### Physical experiments

#### Aerosolisation parameters with Capnopen® and the multi-nozzle nebuliser

We assumed that the multi-nozzle nebuliser would require a higher injection flow and thus a shorter aerosolisation time than Capnopen®. Aerosol generation with a pressure greater than 11 bars is recommended by the manufacturer. The Capnopen® nebuliser reached this pressure within 35 s with an injection flow of 0.6 ml/s, and the multi-nozzle nebuliser reached this pressure within 5 s with an injection flow of 3.4 ml/s. For aerosolisation of 50 ml doxorubicin and 150 ml cisplatin, 5.56 min was required for the Capnopen® nebuliser, and 0.98 min was required for the multi-nozzle nebuliser. The volume of each drug aerosolised below the recommended pressure was 21 ml for Capnopen® and 17 ml for the multi-nozzle nebuliser. As has been demonstrated, the multi-nozzle nebuliser has a shorter aerosolisation time with a sixfold higher injection flow than Capnopen®.

#### Granulometry of aerosol generated with Capnopen® and the multi-nozzle nebuliser

In our experience, smaller and more homogeneous aerosol droplets might increase tissue drug delivery and the homogeneity of tissue drug distribution. The aerosol droplet size and size distribution were measured with distilled water, 5% glucose and 5 cSt silicon oil by laser diffraction spectroscopy. For Capnopen®, the results with distilled water were 34.8 (95% CI 22.8–52.7) µm versus 49.6 (95% CI 28.8–84.8) µm with the multi-nozzle nebuliser; the results with 5% glucose were 39.0 (95% CI 23.7–65.2) versus 42.1 (95% CI 20.5–76.0) µm, and those with 5 cSt silicon oil were 43.0 (95% CI 20.2–78.5) versus 328.7 (95% CI 51.2–662.0) µm, respectively. The aerosol particle size distribution was bimodal with both devices. There were two peaks between 1–10 and 10–100 µm for Capnopen® and between 10–100 and 100–1000 µm for the multi-nozzle nebuliser with 5 cSt silicon oil. Furthermore, the multi-nozzle nebuliser demonstrated a bimodal curve between 1–10 and 10–100 µm with 5% glucose. In short, Capnopen® generated smaller and more homogeneous aerosol than did the multi-nozzle nebuliser (Fig. 2C).

#### Spatial spray patterns with Capnopen® and the multi-nozzle nebuliser

Broader and more homogeneous aerosolisation was expected from the multi-nozzle nebuliser versus Capnopen®. The spray patterns were analysed among three circle zones by ImageJ® after aerosolising blue ink on conic folded blotting paper (Fig. 2B2). The spray patterns of the multi-nozzle nebuliser were evaluated with and without axial rotation (Fig. 2A). The relative integrated staining density after aerosolisation with Capnopen® was 22.6% (95% CI 19.1–26.2), 37.6% (95% CI 36.6–38.6) and 39.8% (95% CI 35.7–43.8) in zones 1, 2 and 3, respectively (post hoc test zone 1 vs. 2 and 1 vs. 3, both *p* < 0.001); for the multi-nozzle nebuliser without axial rotation, the relative integrated staining density in zones 1, 2, and 3 were 17.9% (95% CI 15.2–20.5), 33.4% (95% CI 31.6–35.1) and 48.8% (95% CI 46.1–51.5), respectively (post hoc test, *p* < 0.001); and for the multi-nozzle nebuliser with axial rotation, they were 13.6% (95% CI 12.0–15.3), 29.4% (95% CI 27.2–31.7) and 57.0% (95% 55.0–59.0), respectively (post hoc test, *p* < 0.001) (Fig. 2B1). Capnopen® resulted in greater staining in zones 1 and 2, and the multi-nozzle nebuliser resulted in greater staining in zone 3 (post hoc test, *p* < 0.001). Axial rotation of the multi-nozzle nebuliser further enhanced staining in zone 3 (post hoc test, *p* < 0.001). In summary, Capnopen® presented greater homogeneity of aerosolisation, but the surface ink cover was lower than that of the multi-nozzle nebuliser.

### Ex vivo pharmacological experiments with the eIBUB model

#### Real-time tissue drug uptake

Drug delivery is characterised by weight gain in the target tissue. The eIBUB model allows real-time drug delivery measurement based on the target tissue weight. PIPAC with doxorubicin and cisplatin was conducted using the ex vivo eIBUB model. After PIPAC with Capnopen®, the weight of the target tissue increased to 163.1 ± 20.5% versus 177.3 ± 27.5% with the multi-nozzle nebuliser (one-way ANOVA, *p* = 0.073). The tissue liquid saturation was sixfold quicker with the multi-nozzle nebuliser vs with Capnopen® (one-way ANOVA, *p* = 0.033) (Fig. 3A). Overall, the multi-nozzle nebuliser delivered drugs quicker than Capnopen®. However, the delivered drug volume was the same.

#### Real-time aerosol sedimentation

Aerosol sedimentation demonstrates the effectiveness of drug delivery based on the volume of drugs turned into a liquid after aerosolisation without reaching a target tissue. The less aerosol sedimentation there is, the greater the drug delivery. The eIBUB model allows measurements of sedimented aerosols. After PIPAC with Capnopen®, 47.6 ± 8.0%% of the aerosolised liquid was sedimented, and with the multi-nozzle nebuliser 48.9 ± 21.2% of the aerosolised liquid was sedimented (one-way ANOVA, *p* = 0.788). Aerosol sedimentation occurred mainly during aerosolisation in both groups (one-way ANOVA, *p* = 0.316) (Fig. 3B). Given these points, the multi-nozzle nebuliser did not reduce aerosol sedimentation, showing an approximately threefold greater data deviation than Capnopen®.

#### Tissue drug distribution

A higher tissue drug concentration with a more homogeneous distribution was expected after PIPAC with the multi-nozzle nebuliser. The concentrations of doxorubicin and cisplatin were measured in tissue biopsies taken from the top, middle and bottom of eIBUB tissue after PIPAC with Capnopen® and the multi-nozzle nebuliser. The total doxorubicin concentrations after PIPAC with Capnopen® and the multi-nozzle nebuliser were 1.5 ± 1.2 and 1.3 ± 1.2 ng/mg, respectively (one-way ANOVA, *p* = 0.473) (Fig. 3C), and the cisplatin concentrations were 23.4 ± 9.2 and 14.4 ± 6.3 ng/mg, respectively (one-way ANOVA, *p* < 0.001) (Fig. 3D). The tissue doxorubicin distribution was more homogeneous among the top, middle and bottom of the eIBUB model with the multi-nozzle nebuliser (post hoc, *p* > 0.05) but not significantly different from that of Capnopen® (one-way ANOVA, *p* > 0.05) (Fig. 3E). However, the cisplatin tissue concentration was significantly greater with Capnopen® in the top and middle of the eIBUB model (one-way ANOVA, *p* < 0.05), with a more homogeneous distribution among all areas (post hoc, *p* > 0.05) (Fig. 3F). Overall, the multi-nozzle nebuliser did not increase the tissue drug concentration; higher homogeneity was reached for doxorubicin with the multi-nozzle nebuliser, but not significantly compared to Capnopen®. Furthermore, the cisplatin tissue concentration was significantly greater and more homogeneous after PIPAC with Capnopen® than after PIPAC with the multi-nozzle nebuliser.

## Discussion

This study provided preclinical data for a PIPAC multi-nozzle nebuliser compared to a clinical reference one-nozzle nebuliser, Capnopen®. The performance of both nebulisers was evaluated via physical and ex vivo experiments.

In physical experiments, the multi-nozzle nebuliser showed a sixfold shorter aerosolisation time than Capnopen®, which reduces PIPAC application and surgery time, minimising patient burden and costs. However, a higher velocity of aerosolisation might increase the aerosol density, leading to oversaturation of the environment and causing increased sedimentation and liquid formation [9, 13], which has a less homogeneous distribution and lower penetration depth than aerosols [1–3]. Moreover, the aerosol floating time might play an essential role in tissue drug distribution, especially in the most hidden areas of the abdominal cavity [7]. Although much attention has been given to developing nebulisers with a smaller median aerosol droplet size [14, 15], the particle size distribution is often underestimated. Capnopen® demonstrated a smaller aerosol particle size distribution than did the multi-nozzle nebuliser. Reducing the range between the smallest and the largest aerosol particles can increase the aerosol homogeneity, leading to higher aerosol stability, lower sedimentation, a longer aerosol floating time and thus more homogeneous drug distribution.

Another parameter that influences drug distribution is the angle of aerosolisation. Compared to the one-nozzle Capnopen®, the static and axial rotating multi-nozzle nebuliser demonstrated a more extensive surface cover with ink aerosolised on blotting paper. Similar results were shown with the four-nozzle nebuliser QuattroJet™, where the surface cover was 10–18 times greater than that with the one-nozzle nebulisers 770–12, HurriChem™ and MCR-4 TOPOL® [16]. These data should be carefully considered because even homogeneous surface cover with therapeutics does not guarantee homogeneous tissue drug delivery. This makes preclinical pharmacological distribution studies on ex vivo peritoneal models crucial in developing drug delivery systems.

Here, we investigated the homogeneity of tissue drug distribution in ex vivo eIBUB model. Despite more extensive surface exposure with the multi-nozzle nebuliser, there was no significant difference in tissue drug delivery or aerosol sedimentation compared to Capnopen®, which confirms that there is no correlation between broader drug surface cover and greater tissue drug delivery. Moreover, the cisplatin tissue concentration was significantly greater with Capnopen® with no vertical gradient, which shows that the angle of aerosolisation might not be the only parameter determining tissue drug delivery.

Generating smaller and more homogeneous aerosols can explain the pharmacological advantages of Capnopen®. To our knowledge, the aerosol particle size distribution is multimodal among the most clinically implemented PIPAC nebulisers. However, the smallest fraction is predominantly responsible for better tissue drug distribution, mainly in the most hidden areas. By reducing the particle size from 100 to 1 µm, the aerosol floating time increases 9000-fold [17], which might indeed improve the drug surface exposure. Moreover, the velocity of aerosol particles increases with decreasing particle size driven by Brownian motion, increasing impaction and interception. A multi-nozzle nebuliser oversaturates the environment with unstable aerosol, accelerating sedimentation. When aerosol particles increase in size to 100 µm, they do not behave as an aerosol. In particular, their pathway is mainly defined by the angle of aerosolisation and gravity leading to further enlargement, impaction and liquid formation.

The demonstrated results of tissue drug delivery and aerosol sedimentation were obtained with the ex vivo eIBUB model. Although this model is verified and validated for PIPAC pharmacological experiments on the peritoneum, many anatomical and physiological differences exist compared to in vivo animal models and humans. For example, the absence of blood supply in the eIBUB model leads to tissue necroses and cell membrane damage affecting transmembrane transport. To minimise the post-mortem changes, the organs for the eIBUB model were delivered on ice immediately after explantation, followed by histological screening to exclude any tissue damage and necrosis signs.

Another limitation of the ex vivo eIBUB model is missing tissue drug clearance, modifying tissue drug concentrations and distribution in vivo. For the same reasons, no pharmacokinetic and pharmacodynamic studies are possible. However, the peritoneal blood supply requires only 1–2% of the total systemic flow; hence, no considerable tissue drug clearance is expected [18]. This assumption is also supported by the low systemic toxicity demonstrated in the clinical studies on PIPAC [19].

Although the listed factors play a meaningful role in tissue drug delivery and distribution, their influence could not be considered in our ex vivo study. Nonetheless, as both nebulisers were evaluated under the same conditions and with the same models, there are no concerns about data comparability. Furthermore, the validation of the eIBUB model demonstrated tissue drug distribution near the same as in the human peritoneum, allowing results to be translated into clinical practice. Indeed, in vivo functional studies might be required after ex vivo optimisation of tissue drug delivery to evaluate the toxicity and treatment response.

As mentioned above, the technical characteristics of the nebulisers also determine tissue drug delivery. Although the multi-nozzle nebuliser did not show any advantages over Capnopen®, there are many possibilities for further improvements. We assume a lower velocity of drug aerosolisation with more homogeneous aerosol might prevent the oversaturation of the environment and minimise aerosol sedimentation, leading to more homogenous drug distribution and higher tissue drug concentrations. However, such a device would require lower injection flow, which might decrease the angle of aerosolisation and increase the aerosol particle size, negatively affecting tissue drug delivery. Considering all this, the multi-nozzle nebuliser needs further technological improvements offering a compromise among pressure, injection flow, aerosol particle size and angle of aerosolisation.

From our perspective, engineering the next-generation one-nozzle Capnopen® with a broader angle of aerosolisation and more homogeneous aerosol, e.g. based on the impaction technology, might be more advantageous in tissue drug delivery because a greater angle of aerosolisation and more homogenous aerosol are possible to achieve without a massive increase in aerosolisation velocity or pressure.

## Conclusion

The use of a multi-nozzle nebuliser did not meet expectations. The increased number of spray nozzles did not improve tissue drug delivery or the homogeneity of tissue drug distribution.
